# Management of acute type A aortic dissection with acute lower extremities malperfusion

**DOI:** 10.1186/s13019-019-1033-5

**Published:** 2019-11-27

**Authors:** Dong Hoon Kang, Jong Woo Kim, Sung Hwan Kim, Seong Ho Moon, Jun Ho Yang, Jae Jun Jung, Hyun Oh. Park, Jun Young Choi, In Seok Jang, Chung Eun Lee, Jong Duk Kim, Joung Hun Byun

**Affiliations:** 1 Department of Thoracic and Cardiovascular Surgery, Gyeongsang National University School of Medicine and Gyeongsang National University Changwon Hospital, Changwon, Republic of Korea; 2 Department of Thoracic and Cardiovascular Surgery, Gyeongsang National University School of Medicine and Gyeongsang National University Jinju Hospital, Jinju, Republic of Korea

**Keywords:** Aortic dissection, Cardiopulmonary bypass, Axillo-bifemoral bypass

## Abstract

**Background:**

Acute type A aortic dissection complicated by malperfusion is a life – threatening emergency. The optimal management strategy for malperfusion remains controversial.

**Case presentation:**

A 46-year-old man presented to another institution with acute type A aortic dissection with abdominal aorta occlusion. Motor and sensory grade of both lower extremities were zero. Immediate antegrade distal perfusion of both lower extremities was achieved, and total arch replacement with left axillo-bifemoral bypass was performed. At the time of discharge, motor and sensory grades of both lower extremities were 2 and 3, respectively.

**Conclusion:**

This case demonstrates many of the techniques in the management of acute type A aortic dissection with abdominal aorta occlusion. In this case, direct antegrade perfusion of both lower extremities and axillo-bifemoral bypass may be helpful for patients presenting with severe malperfusion of both lower extremities with acute type A aortic dissection.

## Background

Acute type A aortic dissection complicated by malperfusion is a life – threatening emergency. Many surgeons have advocated for the restoration of true lumen blood flow first. However,the optimal management for malperfusion remains controversial. We report the case of successful management for patient with acute type A aortic dissection complicated by malperfusion by direct antegrade perfusion of both lower extremities and axillo-bifemoral bypass.

## Case presentation

**Fig. 1:**
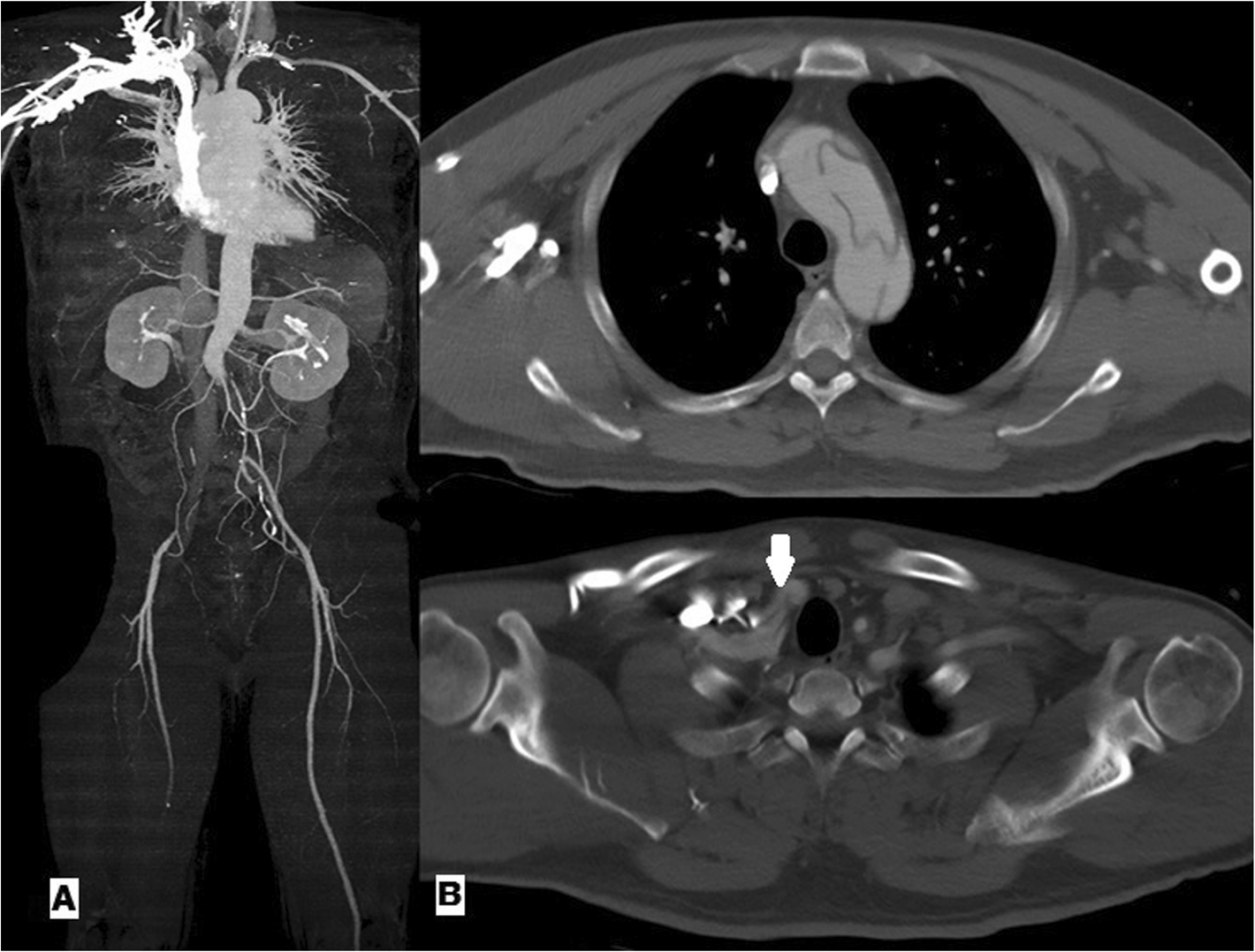
**a**: 3-Dimensional reconstructed aortic CTA showing occlusion of the infra-renal aorta. **b**: Acute type A aortic dissection involving the aortic arch and innominate artery (white arrow). CTA,computed tomographic angiography

A 46-year-old man presented with chest pain and acute paraplegia with acute type A aortic dissection,3 h prior admission. He had no known relevant medical history. Transthoracic echocardiography revealed normal left ventricular function and mild aortic regurgitation. Motor and sensory grades of both lower extremities were zero and pulses of both femoral arteries were absent. Figure 1 shows preoperative aorta computed tomographic angiography (CTA).

**Fig. 2:**
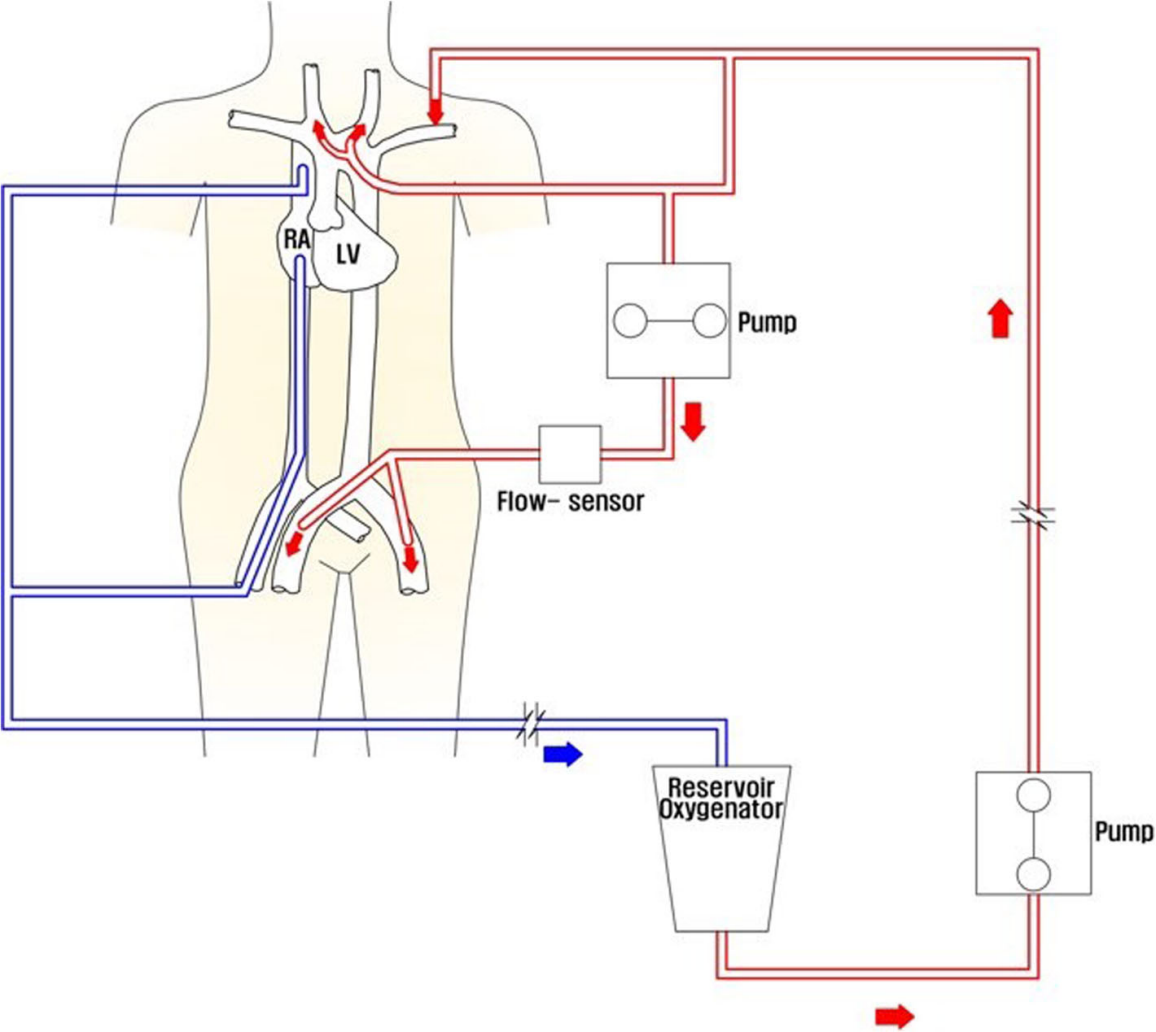
The CPB circuit. CPB,cardiopulmonary bypass

We decided to perform surgery as soon as possible. Figure 2 shows the cardiopulmonary bypass (CPB) circuit. Partial CPB was established (blood flow 1000 cc/min) after insertion of two 14-Fr DLP® arterial cannulas (Medtronic Inc., Minneapolis,MN) via both common femoral arteries for antegrade distal perfusion of both lower extremities as well as 24-Fr venous cannula (Edwards Lifescience LLC, Irvine, CA) via the right common femoral vein. The left axillary artery was used for arterial cannulation using the side graft technique with a 10-mm Dacron graft (Atrium Medical Corporation,Hudson, NH) because of dissection of the innominate artery. Total arch replacement was performed by establishing routine CPB with systemic circulatory arrest (rectal temperature 26 °C) and bilateral antegrade selective cerebral perfusion. During systemic circulatory arrest, perfusion of both lower extremities was maintained.

**Fig. 3:**
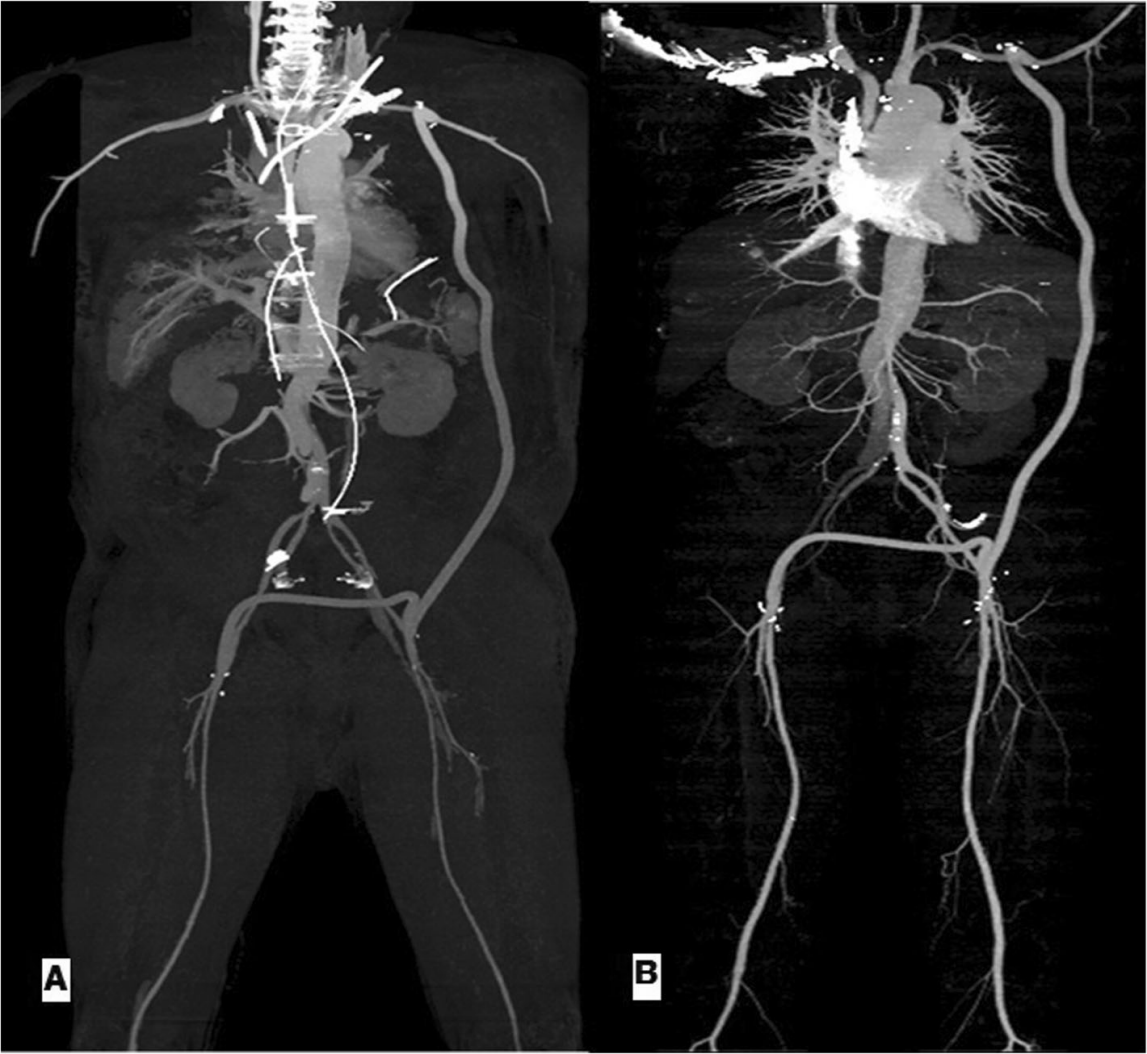
Patent left axillo-bifemoral bypass graft on follow- up aortic CTA (**a**: postoperative 3 days, **b**: postoperative 6 months). CTA, computed tomographic angiography

Maintaining partial CPB for right lower extremity perfusion (blood flow 500 cc/min), left- sided axillo-femoral bypass with an 8 mm Dacron graft (Atrium) was performed. The times for total CPB, aortic cross clamp and systemic circulatory arrest were 320 min, 175 min and 40 min, respectively. In turn, terminating the CPB, femoro-femoral bypass with an 8 mm Dacron graft (Atrium) was performed. At the time of discharge, motor and sensory grades of both lower extremities were 2 and 3, respectively. Figure 3 shows the follow- up aorticCTA.

## Discussion

Acute type A aortic dissection complicated by malperfusion is a life – threatening emergency with perioperative mortality reported in the range of 29 to 89% [1–3]. Early diagnosis is very important for determining management modality. In this situation, many surgeons have advocated for the restoration of true lumen blood flow first. Techniques of fenestration have been developed to restore true lumen blood flow, nevertheless, the ideal management for malperfusion remains controversial [3]. Chiu et al. suggest that operative delay to perform fenestration would not have helped most patients with malperfusion [3]. We agree with this opinions. Especially in the context of ongoing end-organ ischemia, immediate surgery is more appropriate than restoration of true lumen blood flow by fenestration first.

In our case, ischemia time of both lower extremities was about 5 h and the restoration of true lumen blood flow was uncertain with the systemic perfusion via the axillary artery. Therefore, we performed antegrade distal perfusion of both lower extremities first.

Holland et al. reported that the mean flow in four arteries in the leg was 284 ± 21 mL/min in the common femoral artery [4]. In fact, the amount of blood needed for perfusion of both lower extremities was thought to be more, therefore, perfusion was performed at about 500 cc/min each. We performed antegrade distal perfusion with direct cannulation of both femoral arteries for even perfusion of both lower extremities.

Because we planned to perform left axillo-bifemoral bypass to resolve the malperfusion caused by static occlusion of the infra-renal aorta, the left axillary artery was used for CPB. Masashi et al.reported that the left axillary route is preferred over the right, because the left subclavian artery has a separate and downstream origin from the carotid artery [5].

Slonim et al. reported that percutaneous balloon fenestration of the intimal flap and endovascular stenting is an effective treatment for life-threatening ischemic complications of acute aortic dissection. Of the patients,14 patients were treated with stenting and fenestration, 24 with stenting alone, and 2 with fenestration alone [6]. However, we think, in the context of static malperfusion, restoration of true lumen blood flow may not be sufficient by eliminating the tear with the ascending or arch replacement and fenestration. If the size of the preoperative or postoperative fenestration is not appropriate, there may be ongoing false lumen pressurization resulting in persistent malperfusion. In that rationale, we thought, in our case, restoration of true lumen blood flow by eliminating the tear or fenestration would not sufficient, therefore, we performed immediate left axillo-bifemoral bypass after total arch replacement.

## Conclusion

Direct antegrade perfusion of both lower extremities and axillo-bifemoral bypass may be helpful for patients presenting with severe malperfusion of both lower extremities with acute type A aortic dissection.

## Data Availability

Data sharing not applicable to this article as no datasets were generated or analysed during the current study.

## Abbreviations

CPB: cardiopulmonary bypass
CTA: computed tomographic angiography

## References

[1] Girdauskas E, Kuntze T, Borger MA, Falk V, Mohr FW (2009). Surgical risk of preoperative malperfusion in acute type a aortic dissection. J Thorac Cardiovasc Surg.

[2] Deeb GM, Williams DM, Bolling SF, Quint LE, Monaghan H, Sievers J (1997). Surgical delay for acute type a dissection with malperfusion. Ann Thorac Surg.

[3] Chiu P, Tsou S, Andrew B, Goldstone AB, Louie M, Woo YJ (2018). Immediate operation for acute type a aortic dissection complicated by visceral or peripheral malperfusion. J Thorac Cardiovasc Surg.

[4] Holland CK, Brown JM, Scoutt LM, Kenneth JW, Taylor KJW (1998). Lower extremity volumetric arterial blood flow in normal subjects. Ultrasound Med Biol.

[5] Kano M, Chikugo F, Shimahara Y, Urata M, Hayamizu T (2018). Left axillary artery perfusion in surgery of type a aortic dissection. Ann Thorac Cardiovasc Surg.

[6] Slonim SM, Miller DC, Mitchell RS, Semba CP, Razavi MK, Dake MD (1999). Percutaneous balloon fenestration and stenting for life-threatening ischemic complications in patients with acute aortic dissection. J Thorac Cardiovasc Surg.

